# Mind-Body Health Benefits of Traditional Chinese *Qigong* on Women: A Systematic Review of Randomized Controlled Trials

**DOI:** 10.1155/2021/7443498

**Published:** 2021-09-14

**Authors:** Kin-Chung Wilson Leung, Yi-Jian Yang, Stanley Sai-Chuen Hui, Jean Woo

**Affiliations:** ^1^Department of Sports Science and Physical Education, Faculty of Education, The Chinese University of Hong Kong, Hong Kong, China; ^2^CUHK Jockey Club Institute of Ageing, The Chinese University of Hong Kong, Hong Kong, China; ^3^Department of Medicine and Therapeutics, Faculty of Medicine, The Chinese University of Hong Kong, Hong Kong, China

## Abstract

Most women live with an inactive lifestyle, which suggests a need for preference-based choices to promote their participation in physical activity. This systematic review synthesized key findings on the health benefits of *Qigong* among women. We conducted a systematic search of randomized controlled trials (RCTs) of *Qigong* among women according to the PRISMA guidelines using the following databases from their inception through March 2021: PubMed/MEDLINE, Web of Science, Cochrane Library, and US National Library of Medicine. The risk of bias was examined using the Cochrane Collaboration's tool for assessing the risk of bias in randomized trials. Altogether, 18 RCTs were included for final review. Results showed that *Qigong* was a feasible exercise in improving health outcomes, particularly depressive symptoms (63% of trials), quality of life (43%), and fatigue (29%), among general women, intimate partner violence survivors, and women with chronic conditions (e.g., breast cancer patients or survivors). Almost 90% (7/8) of trials reported high adherence rates ranging from 73 to 95% for supervised group training and 63 to 80% for home self-practice. Thus far, there was no evidence of serious adverse effects from performing *Qigong*. For the risk of bias across trials, a lack of allocation concealment (72% of trials), no blinding of participants and personnel (67%), and incomplete outcome data (67%) were the major sources. In summary, *Qigong* is a safe, feasible, and beneficial exercise for general women, abused sufferers, and health-compromised women. However, given the potential risk of bias found in many studies, improved rigor of study design in future trials will be imperatively required.

## 1. Introduction

Despite living longer, women generally have poorer health and lower quality of life than men [1]. This is largely due to complexity of biological and gender-related factors such as violence, early marriage, and inequitable access to healthcare [1]. Increasing evidence suggests that women living with a sedentary lifestyle are vulnerable to disabilities, morbidities, and premature death [2]. A 2018 World Health Organization (WHO) report has shown that the global inactivity prevalence in women (27%) was higher than that in men (20%) [3, 4]. To meet the 2025 global target for inactivity (i.e., 10% reduction) for a healthier world [4], engaging more women in habitual physical activity (PA) to reduce inactivity levels [3] has become a priority in public health.

Several studies have indicated gender differences in PA participation [5–7]. Typically, women are less likely to achieve the WHO-recommended PA levels for health than men throughout the life course [8–11]. From young childhood through late adolescence, a latent class analysis identified three trajectory classes of organized sport participation that were specifically found in boys and girls; “consistent sport participators” for boys (55%) and girls (48%) exhibited the best health profile regarding lean body mass, percent body fat, and perceived physical health [12]. The “sport nonparticipator” class was unique to girls (18%), suggesting that early engagement in organized sport is particularly important in females because they are usually less likely to join sport activities later in life. In middle-aged adults (at the age of 50–65 years), a lower proportion of women (28%) than men (41%) who met the recommended PA guidelines for health was consistently observed [13]. Older women (aged 60 years and older) were also found to participate less in leisure-time PA as measured by both self-reported (−0.8 to −21.4%) and accelerometer-based (–0.2 to –1.5%) methods [10]. Given that marked gender-specific interests in PA participation persist across the lifespan [14, 15], addressing gender preferences for organized activities to enhance PA participation has become a research imperative.

*Qigong* is a traditional Chinese mindfulness-based exercise characterized by an integration of meditation, breathing, interlacing body movements, and coordinated body posture. “*Qi*” (pronounced “chee”) represents the essential energy flowing inside the human body, while “*Gong*” (pronounced “gung”) refers to regular practice in order to cultivate *Qi*. Unlike exercise therapies that only target a specific joint or a series of alternate muscle contraction and relaxation, *Qigong* focuses more on building self-awareness of how the entire body moves through posture and stillness so as to achieve deep states of body-mind relaxation and calmness [16]. Typically, *Qigong* is of lower impact than endurance training and can be done at any level of exercise tolerance, including accommodation for seated or supine exercises [17–20]. Compared to other mind-body exercises (e.g., *Yoga*), which share similar principles (meditation, breath focus, and bodily movements), *Qigong*, a simpler, repetitive form, is believed to be most suitable for women who are less likely to participate in and gain from vigorous activities that require sophisticated skills [15, 21]. For instance, *Baduanjin*, the most common form of *Qigong* (56%) used across 886 primary and secondary research studies [22], is less cognitively demanding than *Yoga* as *Baduanjin* is standardized to have eight sections, while *Yoga* routine is not standardized and has a large variety of movements [23]. Meanwhile, *Baduanjin* requires a lesser extent of physical demands (e.g., balance, joint stretching, and coordinated body movements) than *Yoga* [18, 23]. Other factors that practicing *Qigong* may fascinate women include relief from emotional distress (e.g., chronic psychological stress and depression [24]), weight loss (e.g., fat-burning [25]), improved appearance (e.g., reduced waist circumference [26]), and friendship building (i.e., group exercise) [15, 27, 28]. Thus far, studies found that more than 90% of women practitioners were willing to continue practicing *Qigong* after joining [16, 25, 29], and there were no adverse side effects of *Qigong* reported, even in critically ill patients [20, 30, 31]. However, owing to a lack of public awareness, qualified instructors, and existing dedicated programs [32], *Qigong* seems less familiar than other types of mind-body exercises to Westerners [17].

Despite the fact that recent literature reviews have demonstrated potential benefits of *Qigong* for depressive symptoms [33], body composition [34], and breast cancer [35], which are primary health concerns of women [36–38], a systematic literature review examining women-specific outcomes is still lacking. Besides, most results in recent relevant reviews were obtained from trials varying from randomized controlled trials (RCTs) to quasi-experimental, cohort, and case-control studies [17, 20, 39, 40]. This raises concern over their strength of evidence. A comprehensive review of the benefits of *Qigong* on women can uncover its potential efficacy on women who have multifaceted needs (e.g., emotional support or humanistic care) that are unsolved by conventional therapies. Therefore, the objectives of this systematic review are twofold: (1) to synthesize evidence obtained from RCTs on the health benefits of *Qigong* in women categorized by three major conditions (i.e., general women, abused survivors, and women with chronic conditions) and (2) to describe the risk of bias across the included trials. Our findings may provide insightful ideas for practitioners concerning the development of an effective *Qigong* program that may appeal to different women's motivational profiles.

## 2. Materials and Methods

This systematic review followed the Preferred Reporting Items for Systematic Reviews and Meta-Analyses (PRISMA) guidelines. In line with the PRISMA Checklist [41], the first author developed the study protocol, which was then approved by the coauthors prior to data collection. Reporting of the study flow and findings was in accordance with the PRISMA statement [42].

### 2.1. Inclusion and Exclusion Criteria

All RCTs examining standalone *Qigong* as a major treatment among female participants were included. Due to different physiological bases of external *Qi* therapy that refers to a process by which an experienced *Qigong* practitioner (i.e., acting as a healer) emits his/her *Qi* to clear *Qi* blockage/stagnation, boost *Qi* levels, or balance *Qi* activity of another patient, only supervised or self-practice *Qigong* exercises with meditation and breath focus for enhancing health-related outcomes were considered.

The inclusion criteria were: (1) full text articles, (2) Chinese/English language, (3) peer-reviewed original research, (4) RCT design, and (5) general women, abused survivors, and women with chronic conditions. The exclusion criteria were conference abstracts/reviews, qualitative studies, or studies other than original research such as reviews, meta-analyses, study protocols, comments, letters, case reports, and guidelines.

### 2.2. Databases and Search Terms

Studies published in the databases of PubMed/MEDLINE, Web of Science, Cochrane Library, and US National Library of Medicine were searched from their inception through March 2021. The text word terms used in the database search (title/abstract/subject/keywords) included qigong, qi-gong, qi gong, chi chung, chi gong, chi kung, qi chung, Baduanjin, Ba Duan Jin, Liuziju, Liuzijue, Wuqinxin, Wu Qin Xin, Yijinjing, Yijin Jing, Yi Jin Jing, women, woman, and female. Based on the findings of three recent systematic reviews on *Qigong* and supportive cancer care [17, 20, 31], two text word terms, namely “breast cancer” and “gynecological cancer”, were additionally used for the search. The database search was prerun twice in order to refine the text word terms and strategies (e.g., limiters) used in the search. The final search queries for each database with limiters are summarized in Supplementary Materials.

### 2.3. Article Selection and Review

The eligibility of the searched articles was initially screened based on the title and abstract, followed by the full text. In the initial screening, the searched articles were included and excluded according to the inclusion and exclusion criteria, respectively. Once the information had not been verified in the initial screening, full-text assessment for these studies along with other eligible articles was conducted. To avoid missing any eligible studies, the reference lists of all searched articles or review papers were also screened for additional studies. All duplicates were removed while screening the publications. The data obtained were then summarized into an evidence table, and the coauthors checked and edited each entry for accuracy and consistency. Consensus for relevancy was also made by discussion among the authors.

### 2.4. Data Collection

Study characteristics of the included studies were summarized using a PICO (P: patient, population, and problem; I: intervention; C: comparison; O: outcome) approach. For each study, data on year of publication, country, participant characteristics (e.g., age, health/clinical status, etc.), sample sizes, intervention conditions (e.g., type, frequency, and duration) and logistic (e.g., supervised training or home practice), attrition rates, outcome measures, and key findings were retrieved and tabulated.

### 2.5. Risk of Bias Assessment

The risks of bias of the included studies were examined using the Cochrane Collaboration's tool for assessing the risk of bias in randomized trials [43, 44]. In brief, the tool is composed of seven domains for checking biases: (1) random sequence generation (selection bias), (2) allocation concealment (selection bias), (3) blinding of participants and personnel (performance bias), (4) blinding of outcome assessors (detection bias), (5) incomplete outcome data (attrition bias), (6) selective outcome reporting (reporting bias), and (7) other sources of bias, including the timing of outcome assessment, similarity in randomized groups at baseline, appropriate means for monitoring subject compliance, the appropriate rationale for control groups, fidelity check, sample size estimation, and appropriate judgements for the measurement tools. Studies were classified as having a high risk of bias when one or more bias domains were rated high risk or two or more domains were rated unclear risk. Trials were defined as having a low risk of bias if no bias domain was rated high risk or only one domain was rated unclear risk [45].

## 3. Results

### 3.1. Study Selection

**Figure 1:**
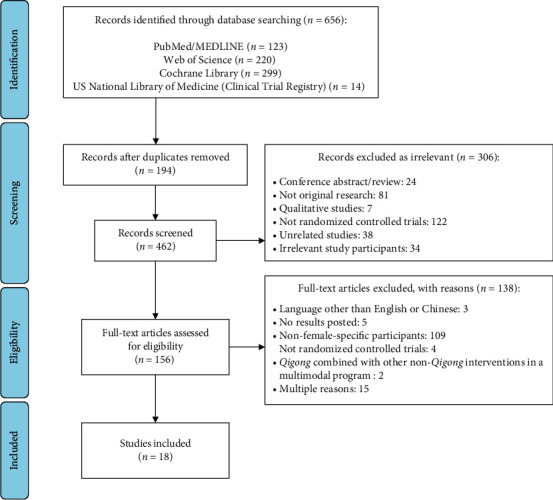
Flow chart of the article selection process.

By March 2021, our database search retrieved 656 records, of which 194 were duplicates. After removing the duplicates, 462 articles were initially screened on their titles and abstracts. The initial screening resulted in 156 articles that were assessed as full text. After the full-text assessment, 138 studies were excluded due to the following reasons: (1) language other than English or Chinese (*n* = 3), (2) no results reported (*n* = 5), (3) non-female-specific participants (*n* = 109), (4) not RCTs (*n* = 4), (5) *Qigong* combined with other non-*Qigong* interventions in a multimodal program (*n* = 2), and (6) multiple reasons (*n* = 15). Finally, a total of 18 studies that met the eligibility criteria were included for review (Figure 1).

### 3.2. Study Characteristics

Characteristics of the 18 included studies are summarized in Table 1. The included studies were published from 1996 to 2020 and conducted in seven places, including China (*n* = 8) [23, 24, 26, 46–50], United States (*n* = 5) [18, 51–54], Australia (*n* = 1) [30], Malaysia (*n* = 1) [55], Spain (*n* = 1) [56], Taiwan (*n* = 1) [57], and Thailand (*n* = 1) [58]. Two studies were published in Chinese [26, 49], and the rest were published in English. The sample sizes ranged from 14 to 271 in total and 8 to 136 in the experimental groups. Of the 18 included studies, 3 (17%) were conducted in general women (i.e., young [56], middle-aged [57], and menopausal [49]), 1 (6%) was carried out in abused women [24], and 14 (78%) were conducted in women with chronic conditions, including breast cancer patients/survivors (*n* = 8) [30, 47, 48, 50–53, 55] or patients with diabetes (*n* = 2) [26, 58], fibromyalgia (*n* = 2) [18, 54], knee osteoarthritis (*n* = 1) [23], and chronic fatigue syndrome (*n* = 1) [46]. *Baduanjin* (*n* = 6) [23, 24, 26, 46, 50, 57] and *Liuzijue* (*n* = 3) [18, 53, 54] were the most commonly used *Qigong*, followed by *Guolin Qigong* (*n* = 2) [47, 48], *Qigong*/*Tai Chi Easy* (*n* = 2) [51, 52], and other *Qigong* styles (*n* = 5) [30, 49, 55, 56, 58]. The intervention durations and supervised training frequency varied from 4 to 24 weeks and 1 to 6 times/week, respectively. Control groups that were commonly used across studies included sham exercise (i.e., mimicking *Qigong* bodily movements without meditation and breath focus; *n* = 5) [18, 51–54], no treatment (*n* = 5) [23, 26, 50, 56, 57], and usual care (*n* = 4) [46, 47, 55, 58].

### 3.3. Potential for Bias

**Figure 2:**
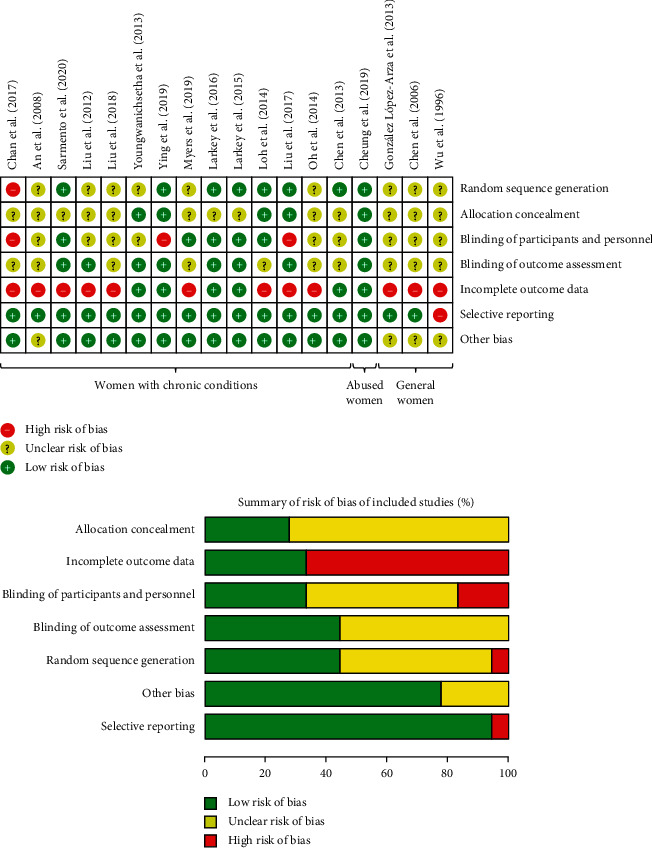
Risk of bias assessment.

All included studies demonstrated some potentials for bias.  Figure 2 summarizes the results of the risk of bias analysis for each study. Overall, 94% (17/18) of the studies had a high or unclear risk of bias in at least one of the seven bias domains [18, 23, 26, 30, 46–58]. Major sources of bias were a lack of allocation concealment (13/18 (72%)) [18, 23, 26, 30, 46, 47, 49, 51–54, 56, 57], no blinding of participants and personnel (12/18 (67%)) [18, 23, 26, 30, 46–50, 56–58], and incomplete outcome data (12/18 (67%)) [18, 23, 26, 30, 46, 48, 49, 53–57]. In addition, only 44% (8/18) of studies clearly described the method of how the random sequence was generated [24, 47, 48, 50–52, 54, 55]. Four studies (22%) did not specify the numbers and reasons for dropouts [26, 46, 49, 57]. Seven studies (39%) reported an attrition rate of 20% or higher [18, 23, 30, 48, 53–55], which is considered as the threshold for significant loss of power in validity [59]. Other biases that may render risk of bias to the results included no details about monitoring of subject compliance throughout the study (4/18 (22%)) [23, 49, 56, 57], no clear description of the rationale for the control groups chosen (12/18 (67%)) [23, 24, 26, 30, 46, 47, 49, 50, 55–58], and no priori sample size estimation for the smallest scientifically meaningful effect size (9/18 (50%)) [18, 23, 26, 30, 46, 49, 54, 56, 57].

## 4. Health Benefits of Qigong on Female Practitioners

### 4.1. General Women

Three trials were conducted in general women (Table 2): (1) young women (aged 18–25 years) [56], (2) middle-aged women (aged 35–60 years) [57], and (3) menopausal women with the climacteric syndrome of durations varying from 4 months to 6 years [49]. The three studies had a high risk of bias (Figure 2).

Wu et al. (1996) [49] showed that climacteric symptoms (e.g., hot flushes, sweating, sleep disturbances, weight gain, mood changes, etc.; *p* < 0.01) and autonomic functions/balance (*p* < 0.05) were improved in menopausal women after practicing *Qigong* versus the drug-treated control group. Serum levels of follicle-stimulating hormone and estradiol were also improved after the intervention (*p* < 0.05).

Chen et al. (2006) [57] showed that *Qigong* could delay bone loss (*p* < 0.049) in middle-aged women by reducing serum levels of interleukin-6 (*p* < 0.001) when compared with no treatment control group.

López-Arza et al. (2013) [56] found that *Qigong* had a significant effect on improving balance (*p* < 0.45) in young women. However, there was no significant difference in the postintervention balance scores between *Qigong* and no treatment control groups.

### 4.2. Abused Women

There was one trial conducted in Chinese women who survived intimate partner violence (IPV) in the past two years [24] (Table 2). The primary outcome was telomerase activity, which is a biomarker of women's cellular longevity that is adversely associated with psychological stress [60]. This study had a low risk of bias (Figure 2).

Cheung et al. (2019) [24] showed that telomerase activity was increased in IPV women after practicing *Qigong* (*p* < 0.05). Ratings of perceived stress and depressive symptoms were improved in both intervention (*p* < 0.001) and wait-list control (*p* < 0.008) groups, yet the between-group difference was only observed after 6-week training (*p* < 0.02) but not upon the completion of the 22-week intervention.

## 5. Women with Chronic Conditions

### 5.1. Breast Cancer Patients/Survivors

Without duplicate counting of participants between Larkey et al. (2015) [52] and Larkey et al. (2016) [51] (i.e., conducted on the same cohort), all trials (*n* = 8) comprised 174 breast cancer patients or survivors (Table 2). Timing of interventions ranged from the early stage in treatment (e.g., receiving active radiotherapy) [47] through the recovery phase after treatment (i.e., survivorship) [48, 50–53, 55]. In one trial, *Qigong* therapy was studied among women with a confirmed diagnosis of metastatic breast cancer [30]. All studies had a high or unclear risk of bias in at least one of the bias domains, and the major source of bias was a lack of allocation concealment (5/8 (63%)) [30, 47, 51–53] (Figure 2). Overall, two trials had a low risk of bias [51, 52], while the other six studies had a high risk of bias [30, 47, 48, 50, 53, 55].

Health effects of *Qigong* among breast cancer patients/survivors were classified according to outcome measures: quality of life (QOL; 6/8 (75%)) [30, 47, 48, 50, 51, 55], fatigue (5/8 (63%)) [30, 47, 52, 53, 55], depressive symptoms (5/8 (63%)) [47, 48, 50, 52, 55], anxiety (3/8 (38%)) [48, 50, 55], sleep (3/8 (38%)) [47, 52, 53], stress (3/8 (38%)) [30, 47, 55], body mass index (BMI; (2/8 (25%)) [50, 51], cognitive function (2/8 (25%)) [51, 53], PA levels (2/8 (25%)) [51, 53], and other outcomes that were reported once, including distress [53], immunological and breast-cancer-specific markers [48], neuropathy [30], sexual function [30], and physical indicators (e.g., arm circumference, shoulder range of motion, cardiopulmonary function, heart rate variability, and lung capacity) [50]. QOL, fatigue, psychological symptoms (anxiety, depressive symptoms, distress, and stress), and sleep disturbances, which were most frequently reported in studies of *Qigong* and supportive cancer care [17, 20, 31, 39, 40, 61], were analyzed as primary outcomes. Other clinical endpoints (e.g., cognitive functions, BMI, PA levels, etc.) were examined as secondary outcomes.

#### 5.1.1. QOL

A self-report instrument, namely the FACT-B (Functional Assessment of Cancer Therapy – Breast; (4/6 (67%)), was most frequently used to examine breast-cancer-specific QOL [30, 48, 50, 55]. QOL was significantly improved in breast cancer survivors after performing *Qigong* versus physical stretching (*p*=0.002) [48], line dancing (*p*=0.036) [55], usual care (*p*=0.048) [55], and no treatment (*p*=0.000) [50]. However, *Qigong* did not significantly improve QOL in metastatic breast cancer patients [30] or patients receiving radiotherapy [47].

#### 5.1.2. Fatigue

Of the five trials that used different measures for fatigue status, one showed a significant effect of *Qigong* versus sham exercise in breast cancer survivors (*p*=0.005) [52]. The other two studies found that *Qigong* was not effective in improving fatigue in breast cancer survivors when compared with line dancing, usual care, sham exercise, or survivorship support [53, 55]. For metastatic breast cancer patients [30] or patients receiving active treatment [47], there was no significant effect of *Qigong* versus control groups, such as meditation or usual care.

#### 5.1.3. Psychological Symptoms

For breast cancer survivors, *Qigong* significantly lowered depression (*p*=0.02) [50] and distress (*p*=0.02) scores [53] in two separate studies when compared with no treatment and survivorship support groups, respectively. For breast cancer patients receiving radiotherapy, *Qigong* was also effective in alleviating depressive symptoms (*p*=0.05) compared to usual care [47]. However, there was no effect of *Qigong* on anxiety [48, 50, 55] and stress [30, 47, 55].

#### 5.1.4. Sleep

There was no significant effect of *Qigong* on sleep quality in either breast cancer patients receiving radiotherapy [47] or survivors [52, 53].

#### 5.1.5. Secondary Outcomes

*Qigong* had a significant effect on improving neuropathy (*p*=0.014) [30], heart rate variability (*p*=0.004) [50], shoulder range of motion on the affected side (*p*=0.000) [50], and immune function (*p*=0.03) [48] when compared with the control groups of meditation, no treatment, and physical stretching. However, there was no effect on PA levels [51, 53], sexual function [30], breast-cancer-specific marker (e.g., serum carcinoma antigen 15-3) [48], arm circumference on the affected side [50], cardiopulmonary function [50], and lung capacity [50]. On the other hand, *Qigong* demonstrated mixed findings on cognitive function [51, 53] and BMI [50, 51].

### 5.2. Diabetic Women

There were two studies conducted in diabetic women: obese [26] and postpartum [58] (Table 2). These two studies had a high risk of bias (Figure 2).

In both obese and postpartum women, *Qigong* had a significant effect on improving fasting plasma glucose (*p* < 0.05) and hemoglobin A1c levels (*p* < 0.05) when compared with a control group of either no treatment or usual care [26, 58]. For postpartum women*, Qigong* was also effective in lowering systolic (*p*=0.016) and diastolic (*p*=0.032) blood pressure [58]. Moreover, *Qigong* had an effect on improving waist circumference or waist-to-hip ratio (*p* < 0.05), blood triglyceride (*p* < 0.05), blood high-density lipoprotein (*p* < 0.05), and serum retinol-binding protein 4 (i.e., a biomarker of glucose metabolism) (*p* < 0.05) in obese diabetic women [26].

### 5.3. Fibromyalgia Patients

Two trials examined the effects of *Liuzijue Qigong* on fibromyalgia symptom management in women patients [18, 54] (Table 2). These two studies had a high risk of bias (Figure 2).

*Qigong* had a significant effect on alleviating fibromyalgia impact (*p* < 0.05), pain (*p* < 0.05), fatigue (*p* < 0.05), sleep disturbances (*p* < 0.05), anxiety (*p* < 0.05), and depression (*p* < 0.05) in fibromyalgia women when compared with sham exercise [18, 54]. However, patients after practicing sham exercise demonstrated a greater QOL improvement (*p* < 0.05) [54].

### 5.4. Other Women Patients

Two studies examined the effects of *Baduanjin Qigong* on knee osteoarthritis [23] and chronic fatigue syndrome [46] (Table 2). These two trials had a high risk of bias (Figure 2).

An et al. (2008) [23] showed that *Qigong* versus no treatment ameliorated pain (*p* < 0.006), stiffness (*p* < 0.029), physical function (*p* < 0.024), aerobic capacity (*p* < 0.036), and peak torque of the knee extensors (*p* < 0.016) in knee osteoarthritis patients.

Chan et al. (2017) [46] demonstrated that *Qigong* was more effective than usual care in improving anxiety (*p* < 0.05) and depressive symptoms (*p* < 0.001) through increased levels of plasma adiponectin (*p* < 0.05) in women with chronic fatigue syndrome.

### 5.5. Adherence to Qigong Programs

Most trials suggested that *Qigong* programs were well tolerated by the participants. Altogether, 7 out of 8 trials (88%) reported adherence rates (on a daily or weekly basis) ranging from 73 to 95% for supervised group training [18, 30, 50, 52, 58] and 63 to 80% for home self-practice [18, 54, 55, 58]. Two studies reported 65–74% of participants attending 80% or more of the supervised training sessions [24, 47]. However, one study demonstrated an adherence rate lower than 60% for both supervised training (52%) and self-practice (31%) sessions [53].

### 5.6. Safety and Adverse Effects

Eight studies indicated that *Qigong* intervention was safe without adverse side effects among various types of women practitioners [18, 23, 24, 30, 47, 51, 54, 58]. Although one study reported that four participants had mild knee pain or shoulder problems after practicing *Qigong*, the symptoms were relieved following action guidance and correction by the instructor [48].

## 6. Discussion

A growing body of studies have examined the potential of mind-body movements to promote health, prevent diseases, and help alleviate disease- or treatment-related symptoms. In the United States, one or more of these mind-body practices comprised up to 30% of exercise programs in fitness centers and health clubs [62, 63], and more women (10.3%; vs. men (5.2%)) applied these movements mainly for the purpose of stress reduction [64]. Empirically, the two key components of these movements, breath focus and mind focus, have been proven potentially beneficial for a variety of health outcomes. Slow and focused deep breathing has been found to stabilize blood pressure [65], oxidative stress [66, 67], and autonomic nervous system [65], while meditation has been mostly associated with improved emotional states (e.g., depression) [68–70]. To the best of our knowledge, the present study is the first systematic review synthesizing knowledge concerning potential health benefits of *Qigong* (a type of mind-body exercise) among women (general women, IPV survivors, and women with chronic conditions), especially in improving depressive symptoms (5/8 (63%)) [24, 46, 47, 50, 54], QOL (3/7 (43%)) [48, 50, 55], and fatigue (2/7 (29%)) [52, 54].

Depressive disorders [71] and fatigue symptoms [72] are affecting more women than men globally, greatly compromising women's overall QOL. *Qigong* has been shown to alleviate the severity of psychological symptoms (e.g., depression and anxiety) likely through modulations of the hypothalamic-pituitary-adrenal axis [73, 74], monoamine neurotransmitter (e.g., serotonin) [73], brain-derived neurotrophic factor [73], and adiponectin [46]. In particular, breast cancer patients often experience substantial treatment side effects that undermine their daily living; improved QOL and fatigue through regularly practicing *Qigong* can have a positive impact on their day-to-day life. Our findings showed that *Qigong* was especially effective in improving QOL and fatigue in breast cancer patients after treatment (i.e., survivorship) [48, 50, 52, 55] but not in women having metastatic cancer [30] or undergoing active radiotherapy [47]. The mixed findings were likely due to persistent psychological distress pertinent to the pretreated disease states, short duration of *Qigong* program (i.e., 5 weeks [47]), or meditation control group used in the study [30]. In fact, meditation practice has been established as an effective complementary medicine for psychological and behavioural disturbances among breast cancer patients during and after treatment [75–78]. This implicates that the health effects of *Qigong* were comparable to other evidence-based mindfulness interventions. As opposed to resistance and aerobic exercise in breast cancer patients whose adherence to supervised training was around 70% and nearly 50% of them attended 80% of the sessions [79, 80], the present review reported a relatively higher adherence rate to supervised *Qigong* programs in breast cancer patients (i.e., 52–95%) [30, 50, 52, 53], of whom more than 65% attended at least 80% of the sessions [47]. This suggests that the tolerance level of supervised *Qigong* programs by women practitioners was comparable or even superior to aerobic and resistance exercise training, especially in breast cancer patients. Thus, we herein proposed that *Qigong* is a feasible, preference-based exercise option for women practitioners.

Despite the health benefits of *Qigong* found in the reviewed studies, improved design in future studies is necessary because many included trials in this review were burdened with a potential risk of bias. The major source was a lack of allocation concealment (>70%) and blinding of participants or personnel (>60%). Across studies with a low risk of bias in allocation concealment, masking of treatment allocation was most commonly performed using opaque envelope technique [24, 48, 50, 58], where a sequentially numbered, sealed, and opaque envelope informing group assignment was distributed to the participants in order to ensure that the investigators who generated random sequence did not know the group allocation results. To further avoid the disclosure of group allocation, Ying et al. (2019) [50] used aluminum foil to make the envelope further invisible, even under intense light. Apart from allocation concealment, blinding of participants from gaining knowledge of *Qigong* should be done at different levels, including study design (e.g., using sham exercise as control [18, 51–54]), subject recruitment (e.g., avoiding words “control/sham”, “intervention/experimental”, or “*Qigong*” in the informed consent [54]), and program implementation (e.g., naming of classes using words other than *Qigong*, such as “rejuvenating movement [51, 52]” or “mild exercise [54]”). However, blinding of participants seems not possible in some scenarios, for example, among patient participants who need to consult family doctors regarding their PA readiness prior to joining exercise programs. To reduce bias for the situation that participant blinding was not feasible, Myers et al. (2019) [53] mentioned that recruitment and consent language should be neutral when describing the intervention and control groups.

To avoid high attrition rates that led to another major source of bias across studies (i.e., incomplete outcome data), Ying et al. (2019) [50] addressed the importance of establishing a rapport with participants in maintaining intervention compliance. Through regular communications by phone, online tools (e.g., WeChat), and face-to-face meetings, participants built a close and warm relationship (like a family) with the researchers involved in the intervention. As a result, only one out of 50 participants withdrew from the intervention due to a vacation trip. Meanwhile, women's PA motivators (e.g., meeting friends, improved appearance, and weight loss) and context preferences (e.g., supervised training, indoor settings, and low cost) should be considered carefully while designing and implementing *Qigong* programs [15]. To address the most common PA barriers associated with traditional gender roles of women in the family (i.e., lack of time due to caregiving burdens [14, 81, 82]), *Qigong* allows home practice for practitioners to preserve travelling time for leisure activities as it does not require any equipment or spacious places to be performed at home. Most importantly, *Qigong*, like *Baduanjin*, is easy to learn via online videos (https://www.youtube.com/watch?v=UJUNfRyAJww) or other media (e.g., instructional DVDs [30, 47, 48, 51]).

Fidelity of interventions is critically essential as practitioners can optimize health benefits derived from performing correct *Qigong* movements, and stay away from undesirable side effects of incorrect practice. As an intervention fidelity check, Larkey et al. (2015 and 2016) [51, 52] examined similarity in the level of exertion and perceived equivalency between *Qigong* and sham exercises (i.e., mimicking *Qigong* bodily movements without meditation and breath focus) in terms of the presence and strengths of breath focus and meditative connection [62]. Obviously, ratings of “breath focus” and “meditative state” were higher in *Qigong* versus sham exercise, which shared similar levels of perceived exertion [52]. The frequency and severity of fatigue among breast cancer survivors were significantly ameliorated when compared with sham exercises. On the principle of traditional Chinese medicine, the underlying mechanism of action is the stimulation of blood and *Qi* circulation, which is a mainstream treatment strategy for chronic fatigue syndrome [83]. Another type of mechanism is the cultivation of a deep sense of relaxation via the focus on breath and meditative states, making *Qigong* more health advantageous than sham exercises. Long-term *Qigong* practice is highly recommended as supported by superior effects of long-term practice (vs. short-term) on fatigued breast cancer patients [52, 53, 55] and healthy adults [84]. Regarding safety, as opposed to a systematic review on exercise therapies showing that nearly 40% of studies reported non-serious adverse events (e.g., pain, fatigue, low back pain, and edema) among participants with or without a medical condition [45], our review showed that only 5% (4/79) of participants in one included study reported recurrence of mild knee pain or shoulder problems from incorrect *Qigong* movements, but the symptoms were quickly relieved after movement correction [48]. With no evidence of serious adverse events (e.g., death, hospitalization, cerebrovascular accident stroke, and hip fractures), *Qigong* seems much safer than conventional exercise therapies, even for end-stage disease patients [30]. Taken together, evaluation of intervention fidelity is very important not only to prevent underestimating the relationship between a *Qigong* intervention and outcome measures but also to avoid drawing false conclusions concerning its effectiveness and safety.

Nonetheless, the findings of this study should be interpreted with caution due to the following limitations. First, a comprehensive meta-analysis was not possibly conducted in the current review as the studies included examined a broad range of outcome measures among a heterogeneity of women practitioners. Second, the validity of our results was challenged by inconsistency in *Qigong* types examined (*Baduanjin*, *Liuzijue, Guolin*, etc.), different types of diseases (e.g., breast cancer, diabetes, fibromyalgia, knee osteoarthritis, or chronic fatigue syndrome), various stages of diseases (e.g., diagnosed with, treated for, and surviving breast cancer), and varying length of interventions. Third, although some studies used the same instrument (i.e., FACT-B) for measuring QOL in breast cancer patients/survivors [30, 48, 50, 55], we were still unable to conclude what dosage of a *Qigong* intervention (e.g., frequency, intensity, and duration) is most appropriate for improving QOL largely owing to the presence of mixed findings. For instance, when compared with control groups, 6-, 8-, and 24-week interventions were effective in improving QOL [30, 48, 55], yet no significant effect was observed in a 10-week intervention [50]. Factors that largely contributed to the mixed findings were different control group comparisons, various diseases stages (e.g., before and after treatment), different sample sizes, and attrition rates. In view of this, we recommend people to practice *Qigong* regularly as supported by foreseeable health benefits of long-term practice [52, 53, 55, 84], and no adverse side effects reported thus far. Fourth, diverse control group design across studies would adversely influence the reliability of our findings. Future research adopting a well-designed control group (e.g., sham-controlled interventions) would further uncover the unique health effects of *Qigong* (e.g., meditative aspects) as opposed to other exercises at similar levels of aerobic exertion. Last but not least, most studies employed subjective measures of health outcomes, which are cognitively reliant and hence give rise to substantive recall bias, especially among older adults or cognitively impaired patients.

## 7. Conclusion

Promoting fitness in women is not an easy task, and this underscores a pressing need for preference-based exercise options in order to facilitate their PA participation. Typically, *Qigong* is of low intensity and easier to adopt than strenuous aerobic or resistance training, thereby favoring women's participation that is likely to be improved with simple exercises in less strenuous formats and without required skills [15, 21]. This systematic review of RCTs further suggests that *Qigong* is an evidence-based exercise and potentially beneficial for women, especially on depressive symptoms [24, 46, 47, 50, 54], QOL [48, 50, 55], and fatigue [52, 54]. Compared with aerobic and resistance training, *Qigong* is more tolerated by women practitioners. This suggests that *Qigong* is a feasible, preferred activity option for women. Amid the COVID-19 pandemic, many people may suffer from physical inactivity due to hospitalization, bed rest, sustained quarantine, and social distancing. For individuals who want to reduce the COVID-associated sedentariness, self-practicing *Qigong* at home is evidently feasible with simple forms of movements, high comprehensibility, and no equipment or spacious places required for maintaining physical fitness and health.

## Figures and Tables

**Table 1:** Characteristics of the included randomized controlled trials published from 1996 to 2020 (*n* = 18).

	Number of study (%)
Language
English	16 (88.9)
Chinese	2 (11.1)

Place	
China	8 (44.4)
United States	5 (27.8)
Australia	1 (5.6)
Malaysia	1 (5.6)
Spain	1 (5.6)
Taiwan	1 (5.6)
Thailand	1 (5.6)

Study participants	
Women with chronic conditions	14 (77.8)
General women	3 (16.7)
Abused women	1 (5.6)

*Qigong* types	
*Baduanjin*	6 (33.3)
*Liuzijue*	3 (16.7)
*Guolin*	2 (11.1)
*Qigong*/*Tai Chi Easy*	2 (11.1)
Others	5 (27.8)

**Table 2:** Summary of randomized controlled trials included in the systematic review (*n* = 18).

	Study participants			Intervention	Control	Attrition rate	Outcome measures	Key findings^Ϯ^
	Health condition	Age (years)						
Author and place		Range	Mean/median					
General women								
Wu et al. (1996), China [49]^¶^	Menopausal	40–60	50	Unspecified *QG* (*n* = 32); 1 month	Treated with vitamins E, B6, and/or oryzanol (n = 18)	QG: 6% Control: 17%	a) Climacteric syndrome (KI) b) Autonomic balance (Wengerǁ) c) Serum FSH (RIA) d) serum LH (RIA) e) serum 2 (RIA) f) serum P (RIA) g) serum T (RIA)	Within-group improvement: (a) *p* < 0.01 b), d), e) *p* < 0.05 c), f), g) No significant changes *QG*/control: a) *p* < 0.01 b) *p* < 0.05
Chen et al. (2006), Taiwan [57]	Middle-aged	35–60	*QG*: 45.7 ± 6.1 Control: 44.6 ± 5.5	*Baduanjin QG* (*n* = 45): 3 times/week; 12 weeks	No treatment (*n* = 45)	*QG*: 2% Control: 4%	a) Serum IL-6 (ELISA) b) BMD (densitometry)	Within-group improvement: a) *p* < 0.001 b) No significant changes *QG*/control: a) *p* < 0.001 b) *p*=0.49
González López-Arza et al. (2013), Spain [56]	Young	18–25	22.4 ± 2.5	*Wang Ziping QG* (*n* = 15); 60 min/session; twice a week; 4 weeks	No treatment (*n* = 15)	*QG*: 13% Control: 7%	a) Balance (UBT)	Within-group improvement: a) *p* < 0.045 *QG*/control: a) No significant differences

Abused women								
Cheung et al. (2019), China [24]	Survivors of intimate partner violence	≥18	*QG*: 42.0 ± 8.7 Control: 41.5 ± 9.3	*Baduanjin QG* (*n* = 136) Week 1–6 (training): 120 min/session; twice a week; 6 weeks Week 7–22: 60 min/session; once a week; 16 weeks Home practice: 30 min/day; once daily, 22 weeks	Wait-list with health education (*n* = 135)	*QG*: 12% Control: 6%	a) PBMC telomerase activity (ELISA) b) Plasma TNF (ELISA) c) Plasma IL-6 (ELISA) d) Depression (BDI) e) Stress (PSS) f) Coping (PCS)	Within-group improvement: a) *p*=0.05 d), e) *p* < 0.001 *QG*/control: d), e) 6-week: *p*=0.009 and 0.02, respectively; 22-week: No significant differences a), b), c), f) No significant differences

Women with chronic conditions								
Chen et al. (2013), China [47]^⸸^	Breast cancer patients receiving radiotherapy	25–64	*QG*: 45.3 ± 6.3 Control: 44.7 ± 9.7	*Guolin QG* (*n* = 49); 40 min/session; once a week; 5 weeks	Wait-list with usual care (*n* = 47)	*QG*: 2% Control: 8%	a) QOL (FACT-G) b) Depression (CES-D) c) Fatigue (BFI) d) Sleep quality (PSQI) e) Stress/cortisol rhythm (TRFIA)	Within-group improvement: b) *p*=0.001 a), c), d), e) No significant changes *QG*/control: b) *p*=0.05 a), c), d), e) No significant differences Higher baseline depressive group: a) *p* < 0.01 b) *p* < 0.06 c) *p* < 0.05 d), e) No significant differences
Oh et al. (2014), Australia [30]	Metastatic breast cancer	≥18	*QG*: 56.9 ± 12.1 Control: 57.8 ± 10.8	Medical *QG* (*n* = 14); 60 min/session; once a week; 10 weeks Home practice: 15–30 min/session; 3–4 times/week; 10 weeks	Meditation (*n* = 13)	*QG*: 36% Control: 38%	a) QOL (FACT-B) b) Fatigue (FACT-F) c) Stress (PSS) d) Neuropathy (FACT-GOG-NTX) e) Sexual function (SFQ)	*QG*/control: d) *p*=0.014 a), b), c), e) No significant differences
Liu et al. (2017), China [48]	Breast cancer survivors	21–80	*QG*: 50.9 ± 7.0 Control: 51.3 ± 7.3	*Guolin QG* (*n* = 79); 60 min/session; twice a week; 24 weeks Home practice: 40 min/session; 5 times/week; 24 weeks	Physical stretching (*n* *=* 75)	*QG*: 41% Control: 18%	a) QOL (FACT-B) b) Anxiety (HADS) c) Depression (HADS) d) Immunological markers (serum IL-2, IFN-*γ*, and TNF) (ELISA) e) Breast cancer-specific marker (serum CA 15–3) (RIA)	Within-group improvement: a), c) *p* < 0.05 b) *p* < 0.01 d) All markers: *p* < 0.001 e) *p* < 0.001 *QG*/control: a) *p*=0.002 d) IFN-*γ* and TNF: *p*=0.03 b), c), e) No significant differences
Loh et al. (2014), Malaysia [55]	Breast cancer survivors	18–65	n/a	*Zhi Neng QG* (*n* = 66): 90 min/session; once a week; 8 weeks Home practice: 30 min/session; twice a week; 8 weeks	(i) Line dancing (*n* = 65) (ii) Wait-list with usual care (*n* = 66)	*QG*: 52% Line dancing: 52% Usual care: 52%	a) QOL (FACT-B) b) Fatigue (FACIT-F) c) Depression (DASS-21) d) Anxiety (DASS-21) e) Stress (DASS-21)	*QG*/Line dancing: a) *p*=0.036 b), c), d), e) No significant differences *QG*/usual care: a) *p*=0.048 b), c), d), e) No significant differences
Larkey et al. (2015), United states [52]	Breast cancer survivors	40–75	*QG*: 57.7 ± 8.9 Control: 59.8 ± 8.9	*QG*/*TCE*^§^ (*n* = 49); 60 min/session; 1–2 times/week; 12 weeks Home practice: 30 min/session; 5 times/week; 12 weeks	Sham *QG*^ǂ^ (*n* = 52)	*QG*: 14% Control: 13%	a) Fatigue (FSI) b) Depression (BDI) c) Sleep quality (PSQI)	Within-group improvement: b), c) *p* < 0.05 *QG*/control: *a) p* = 0.005 at postintervention; *p* = 0.024 at 3-month follow-up b), c) No significant differences
Larkey et al. (2016), United States [51]	Breast cancer survivors	40–75	*QG*: 57.7 ± 8.9 Control: 59.8 ± 8.9	*QG*/*TCE*^§^ (*n* = 49); 60 min/session; 1–2 times/week; 12 weeks Home practice: 30 min/session; 5 times/week; 12 weeks	Sham *QG*^ǂ^ (*n* = 52)	*QG*: 14% Control: 13%	a) Mental and physical QOL (SF-36) b) Cognitive function (FACT-COG (subjective) and WAIS-III (objective)) c) PA levels (BPAQ) d) BMI	Within-group improvement: a) *p* < 0.001 b) FACT-COG: *p* < 0.001; WAIS-III: *p* < 0.05 c) *p*=0.015 d) *p*=0.048 *QG*/control: d) *p*=0.048 a), b), c) No significant differences 3-month follow-up: all with no significant changes across time or intergroup differences
Myers et al. (2019), United States [53]	Breast cancer survivors	>18	*QG*: 52.9 ± 12.0 Sham *QG*: 53.1 ± 10.7 SS: 56.2 ± 11.3	*Liuzijue QG* (*n* = 19); 60 min/session; once a week; 8 weeks Home practice: 15 min/session; twice daily; 8 weeks	(i) Sham *QG*^ǂ^ (*n* = 20) (ii) Survivorship support (SS) (*n* = 11)	*QG*: 21% Sham *QG*^ǂ^: 50% SS: 0%	a) Cognitive function (FACT- COG (subjective), RAVLT, and TMT (objective)) b) Fatigue (MDASI) c) Sleep disturbance (MDASI) d) Distress (MDASI) e) PA levels (WHI PAQ)	*QG*/sham: a) TMT: *p*=0.007 b), c), d), e) No significant differences *QG*/SS: a) FACT-COG: *p* < 0.05 d) *p* < 0.02 b), c), e) No significant differences
Ying et al. (2019), China [50]	Breast cancer survivors	36–72	54.1 ± 7.8	*Baduanjin QG* (*n* = 50): 60 min/session; 3 times/week; 6 months Home practice: 20 min/session; 4 times/week; 6 months	No treatment (*n* = 50)	*QG*: 8% Control: 20%	a) BMI b) Heart rate variability (heart rate monitor) c) Lung capacity (spirometry) d) Arm circumference on the affected side e) Shoulder ROM on the affected side (goniometry) f) Cardiopulmonary function (3-min step test) g) Anxiety (GAD-7) h) Depression (PHQ-9) i) QOL (FACT-B)	*QG*/control: b) *p*=0.004 e), i) *p*=0.000 h) *p*=0.020 a), c), d), f), g) No significant differences

Youngwanich setha et al. (2013), Thailand [58]	Diabetic, postpartum	n/a	*QG*: 35.0 ± 5.6 Control: 36.2 ± 4.5	*Lin Housheng QG* (*n* = 34) Week 1 and 2: 50 min/session; 3 times/week; 2 weeks Week 3–12: Home practice: 5 times/week; 10 weeks	Usual care (*n* = 35)	*QG*: 6% Control: 9%	a) FPG (chemical analyzer) b) HbA1c (chemical analyzer) c) Blood pressure d) Body weight, BMI	*QG*/control: a) *p*=0.018 b) *p*=0.038 c) Systolic: *p*=0.016; diastolic: *p*=0.032 d) No significant differences
Liu et al. (2018), China [26]^¶^	Diabetic, obese	n/a	57.2 ± 5.4	*Baduanjin QG* (*n* = 20): 90 min/session; 6 times/week; 24 weeks (2-week training, followed by 22-week self-practice)	No treatment (*n* = 20)	*QG*: 15% Control: 10%	a) Body weight, BMI b) WC, WHR c) FPG (chemical analyzer) d) HbA1c (chemical analyzer) e) TG (chemical analyzer) f) TC (chemical analyzer) g) LDL (chemical analyzer) h) HDL (chemical analyzer) *i*) serum RBP4 (ELISA)	Within-group improvement: b), c), d), e), h), i) *p* < 0.05 a), f), g) No significant changes *QG*/control: b), c), d), e), h), i) *p* < 0.05 a), f), g) No significant differences
Liu et al. (2012), United States [18]	Fibromyalgia	24–70	*QG*: 55.7 Control: 57.5	*Liuzijue QG* (*n* = 8); 45–60 min/session; once a week; 6 weeks Home practice: 15–20 min/session; twice daily; 6 weeks	Sham *QG*^ǂ^ (*n* *=* 6)	*QG*: 25% Control: 0%	a) Pain (SMPQ) b) Fatigue (MFI-20) c) Sleep quality (PSQI) d) Fibromyalgia impact (FIQ)	*QG*/control: a), b), c), d) *p* < 0.0125
Sarmento et al. (2020), United States [54]	Fibromyalgia	18–70	*QG*: 42.6 ± 10.7 Control: 56.1 ± 12.3	*Liuzijue QG* (*n* = 14); 45 min/session; once a week; 10 weeks Home practice: Twice daily; 10 weeks	Sham *QG*^ǂ^ (*n* = 14)	*QG*: 29% Control: 29%	a) Pain (SMPQ, VAS, and PPT) b) Sleep quality (PSQI) c) Fatigue (FIQ) d) Anxiety (HADS) e) Depression (HADS) f) Fibromyalgia impact (FIQ) g) QOL (QOLS)	*QG*/control: *a*), b), c), d), e), f) *p* < 0.05 g) *p* < 0.05 for control group presenting greater improvement
An et al. (2008), China [23]	Knee osteoarthritis	>55	*QG*: 65.4 ± 8.2 Control: 64.6 ± 6.7	*Baduanjin QG* (*n* = 14)*:* 30 min/session; 5 times/week; 8 weeks	No treatment (*n* = 14)	*QG*: 21% Control: 29%	a) Pain (WOMAC) b) Stiffness (WOMAC) c) Physical function (WOMAC) d) Aerobic capacity (6-MWT) e) Peak torque of the knee extensors (Isokinetic dynamometry) f) General health, social function, and mental health (SF-36)	QG/control: a) *p*=0.006 b) *p*=0.029 c) *p*=0.024 d) *p*=0.036 e) *p*=0.016 f) No significant differences
Chan et al. (2017), China [46]	Chronic fatigue syndrome	<50	*QG*: 39.5 Control: 42.0	*Baduanjin QG* (*n* = 46)*:* 90 min/session; 16 sessions; 9 weeks	Wait-list with usual care (*n* *=* 62)	n/a	a) Plasma adiponectin (ELISA) b) Anxiety (HADS) c) Depression (HADS)	QG/control: a), b) *p* < 0.05 c) *p* < 0.001 3-month follow-up: all with no significant differences

6-MWT, 6-minute walk test; BDI, Beck Depression Inventory; BMD, bone mineral density; BMI, body mass index; BFI, Brief Fatigue Inventory; BPAQ, Brief Physical Activity Questionnaire; CA 15-3, carcinoma antigen 15-3, CES-D, Center for Epidemiologic Studies Depression Scale; DASS-21, Depression and Anxiety Stress Scale-21; E2, estradiol; ELISA, enzyme-linked immunosorbent assay; FACIT-F, Functional Assessment of Chronic Illness Therapy-Fatigue; FACT-B, Functional Assessment of Cancer Therapy-Breast; FACT-COG, FACT-Cognitive Function; FACT-F, FACT-fatigue; FACT-G, FACT-General; FACT-GOG-NTX, FACT-Gynecologic Oncology Group-Neurotoxicity; FIQ, Fibromyalgia Impact Questionnaire; FPG, fasting plasma glucose; FSH, follicle-stimulating hormone; FSI, Fatigue Symptom Inventory; GAD-7, generalized anxiety disorder-7; HADS, Hospital Anxiety and Depression Scale; HbA1c, hemoglobin A1c; HDL, high-density lipoprotein; IFN-*γ*, interferon- *γ*; IL-2, interleukin-2; IL-6, interleukin-6; KI, Kupperman Index; LDL, low-density lipoprotein; LH, luteinizing hormone; MDASI, MD Anderson Cancer Symptom Inventory; MFI-20, Multidimensional Fatigue Inventory; P, progesterone; PA, physical activity; PBMC, peripheral blood mononuclear cells; PCS, Perceived Coping Scale; PHQ-9, Patient Health Questionnaire; PPT, pressure pain threshold; PSQI, Pittsburgh Sleep Quality Index; PSS, Perceived Stress Scale; *QG*, *Qigong*; QOL, quality of life; QOLS, Quality of Life Scale; RAVLT, Rey auditory verbal learning test; SF-36, Short Form-36; SFQ, Sexual Functioning Questionnaire; RBP4, retinol binding protein 4; RIA, ROM, range of motion; radioimmunoassay; SMPQ, short-form McGill Pain Questionnaire; *T*, testosterone; TC, total cholesterol; *TCE*, *Tai Chi Easy*; TG, triglyceride; TMT, trail making test; TNF, tumor necrosis factor; TRFIA, time-resolved fluorescence immunoassay; UBT, unipedal balance test; VAS, visual analog scale; WAIS-III, Wechsler Adult Intelligence Scale-Third Edition; WC, waist circumference; WHI PAQ, women's health initiative brief physical activity questionnaire; WHR; waist-to-hip ratio; and WOMAC, Western Ontario and McMaster Universities Osteoarthritis Index. ^¶^Only Chinese version is available. ^⸸^Participants were randomly assigned to either the *QG* or control groups by adaptive randomization, namely “minimization”, so the groups were balanced evenly according to demographic information and clinical conditions. ^§^Tai Chi Easy (*QG*/*TCE*) is a simplified form of traditional Tai Chi, which is repeated and also includes *QG* bodily movements. The adaptations to the pace, the repetition, and the ease of learning make *TCE* like a typical *QG* exercise. ^ǂ^Sham Q*G* mimicked the *QG* movements but without healing sounds or meditation and breath focus. ^ǁ^Autonomic response patterns in resting conditions (i.e., salivary output, systolic and diastolic blood pressure, heart period, respiration period, and sublingual temperature) were examined in order to reflect participants' autonomic balance conditions before and after the intervention. ^Ϯ^All key findings with significant levels demonstrated improvements in the outcome measures, except specifically indicated. “Within-group improvement” represented within-group improvement for specific outcomes for intervention groups from baseline to the completion of the intervention, while “*QG*/control”, “*QG*/line dancing”, “*QG*/usual care”, “*QG*/sham”, and “*QG*/SS” represented between-group differences in either percentage or absolute changes from baseline to the completion of the intervention.

## Supplementary Materials

Supplementary Materials 1. PubMed (MEDLINE) search strategy. 2. Web of Science search strategy. 3. Cochrane Library. 4. US National Library of Medicine (Clinical Trial Registry).
