# Supplementary material for: Mind-Body Health Benefits of Traditional Chinese Qigong on Women: A Systematic Review of Randomized Controlled Trials
Source: Evid Based Complement Alternat Med. 2021 Sep 14;2021:7443498. doi: 10.1155/2021/7443498 (PMC8457943; doi:10.1155/2021/7443498)
Supplement: Supplementary Materials — 1. PubMed (MEDLINE) search strategy. 2. Web of Science search strategy. 3. Cochrane Library. 4. US National Library of Medicine (Clinical Trial Registry). [file 7443498.f1.docx]

**Supplementary Material**

1. **PubMed (MEDLINE) Search Strategy**

(“qigong”[All Fields] OR “qi-gong”[All Fields] OR “qi gong”[All Fields] OR “chi chung”[All Fields] OR “chi gong”[All Fields] OR “chi kung”[All Fields] OR “qi chung”[All Fields] OR “Baduanjin”[All Fields] OR “Ba Duan Jin”[All Fields] OR “Liuziju”[All Fields] OR “Liuzijue”[All Fields] OR “Wuqinxin”[All Fields] OR “Wu Qin Xin”[All Fields] OR “Yijinjing”[All Fields] OR “Yijin Jing”[All Fields] OR “Yi Jin Jing”[All Fields])

AND

(“women”[All Fields] OR “woman”[All Fields] OR “female”[All Fields] OR “breast cancer”[All Fields] OR “gynecological cancer”[All Fields])

**Limiters**

- Text availability: Full text
- Article type: Randomized Controlled Trial
- Species: Humans
- Language: Chinese/English
- Sex: Female

1. **Web of Science Search Strategy**

All Fields = “qigong” OR “qi-gong” OR “qi gong” OR “chi chung” OR “chi gong” OR “chi kung” OR “qi chung” OR “Baduanjin” OR “Ba Duan Jin” OR “Liuziju” OR “Liuzijue” OR “Wuqinxin” OR “Wu Qin Xin” OR “Yijinjing” OR “Yijin Jing” OR “Yi Jin Jing”

AND

All Fields = “women” OR “woman” OR “female” OR “breast cancer” OR “gynecological cancer”

**Limiters**

- Document Types: ARTICLE
- Languages: English

1. **Cochrane library**

Title Abstract Keyword = “qigong” OR “qi-gong” OR “qi gong” OR “chi chung” OR “chi gong” OR “chi kung” OR “qi chung” OR “Baduanjin” OR “Ba Duan Jin” OR “Liuziju” OR “Liuzijue” OR “Wuqinxin” OR “Wu Qin Xin” OR “Yijinjing” OR “Yijin Jing” OR “Yi Jin Jing”

AND

Title Abstract Keyword = “women” OR “woman” OR “female” OR “breast cancer” OR “gynecological cancer”

**Limiters**

- Content type: Trials

1. **US National Library of Medicine (Clinical Trial Registry)**

Other terms = “qigong” OR “qi-gong” OR “qi gong” OR “chi chung” OR “chi gong” OR “chi kung” OR “qi chung” OR “Baduanjin” OR “Ba Duan Jin” OR “Liuziju” OR “Liuzijue” OR “Wuqinxin” OR “Wu Qin Xin” OR “Yijinjing” OR “Yijin Jing” OR “Yi Jin Jing”

**Limiters**

- Study type: All Studies
- Study Results: Studies with Results
- Sex: Studies with Female Participants
